# Effect of Small Nuclear RNA 64 (SNORA64) on Apoptosis Regulator Genes in Pancreatic Cancer in Vitro

**DOI:** 10.12688/f1000research.173746.1

**Published:** 2026-01-16

**Authors:** Rana Alfardan

**Affiliations:** 1 Community Health Techniques Department, Southern Technical University, Basrah, Basrah, 61004, Iraq

**Keywords:** Apoptosis, Pancreatic Cancer, Cellular viability, Regulator genes, SNORA64

## Abstract

**Background:**

Pancreatic cancer has a poor prognosis and is highly aggressive and deadly. Most pancreatic cancer diagnoses are adenocarcinomas, which account for more than 90 % of all cases. Developing effective therapeutic strategies requires an understanding of the molecular mechanisms associated with pancreatic cancer progression. Small nucleolar RNA 64 (SNORA64) was presented as a predictive marker for pancreatic cancer stages in our previous study. SNORA64 showed a gradual loss of its expression throughout the carcinogenesis process, and it inhibited metastasis by interfering with epithelial to mesenchymal transition (EMT). In this study, we investigated the role of SNORA64 on an intrinsic apoptotic pathway in pancreatic cancers by using human pancreatic cell line derived from adenocarcinoma PK-8 with SNORA64 knockdown and the scramble to compare with.

**Methods:**

QPCR techniques used to measure the gene expression level of apoptosis related genes and cell viability analyzer are implanted in this study as investigational methods.

**Results:**

Pk-8 with low expression of SNORA64 shows significantly high expression of anti-apoptotic genes B-cell leukemia/lymphoma-2 (BCl2) and B-cell lymphoma-extra-large (BCL-Xl) in contrast to the scramble control cell line. Conversely, the pro-apoptotic genes BH3 interacting domain death agonist (BID), BCL2 Associated X (BAX), and BCL2 homologous antagonist/killer (BAK) show significantly low expression compared to the scramble control. However, there is no change in the expression of BAD and BIM in Pk-8 with SNORA64 knockdown compared to the scrambled control cell line. Furthermore, the Pk-8 with low expression of SNORA64 shows a significant high proliferation rate and viability percentage compared to the scramble control cell line.

**Conclusion:**

The downregulation of SNORA64 affects apoptosis pathways by manipulating pro- and anti-apoptotic gene regulators. The SNORA64 interactions with apoptotic inhibitor molecules and downregulation of pro-apoptotic molecules significantly sustain cellular viability. Therefore; SNORA64 can be used to increase the cell sensitivity to death during treatment.

## 1. Introduction

This Adenocarcinoma and neuroendocrine pancreatic cancer are two major types of pancreatic cancer. Most pancreatic cancer diagnoses are adenocarcinomas, which account for more than 90 % of all cases and originate in the pancreas’ duct lining. ^1^ There are limited treatment options for pancreatic cancer, because of its aggressive and lethal nature, it has a dismal prognosis. In order to develop effective therapeutic strategies for pancreatic cancer, it is essential to understand the molecular mechanisms involved in its progression. ^2^ Cancer cells, however, are often able to escape cell death and continue to proliferate in pancreatic cancer due to the deregulation of the apoptosis pathway. ^1^ The process of apoptosis, or programmed cell death, maintains tissue homeostasis and prevents cancer triggered by several stimuli. During early development and in pathophysiological conditions, programmed cell death plays a key role in maintaining morphogenetic homeostasis. ^3^ The development and progression of cancer are associated with the deregulation of different apoptotic components. ^2^ Apoptosis requires the activation of distinct signaling pathways that are frequently deregulated by cancer, such as tumor suppressor P53. ^3^ P53 is the most common genetic alteration found in clinical tumor samples, and there’s a positive statistical association between its expression and apoptosis. ^4^ It may therefore be possible to track the progression of cancer by investigating the single or multiple apoptotic components involved in its expression during carcinogenesis. ^2^ DNA damage and oxidative stress are examples of internal, or so-called mitochondrial stimuli that can also affect apoptosis (as well as external stimuli). ^2, 3^ In the intrinsic apoptotic pathway, members of the B-cell leukemia/lymphoma-2 (BCl2) family include groups of genes known as pro- or antiapoptotic proteins. Apoptosis activation is relatively dependent on the balance between the expression of these genes. There are anti-apoptotic regulators (BCl2, BCL-XL, BCLDW, and MCL-1), pro-apoptotic BH3-only regulators that act as apoptotic sensitizers (BAD, NOXA, HRK, BIK, BMF), pro-apoptotic BH3-only regulators that act as apoptotic activators (BID, BIM, and PUMA), and pore-forming regulators that act as effectors (BAX, BAK). ^3^ Specifically, the BCl2 family of proteins is responsible for regulating mitochondrial outer membrane permeabilization. Any change in mitochondrial outer membrane permeabilization leads to the irreversible release of intermembrane space proteins such as Cytochrome c (Cyt.c). ^5^ The latter is a water-soluble protein released from the mitochondrial membrane in the cytoplasm and initiates the executive apoptotic pathways, such as activates the execution pathway mediated by caspase enzymes. Several cancer types have been associated with Cyt.c as a prognostic indicator of apoptosis, based on the data available so far. Researchers discovered that NS398 medications were linked to the suppression of Cyclo-oxygenase-2 (COX-2) production in esophageal cancer cells via a cyt. c-dependent pathway. ^6^ In a similar study, teniposide increased Cyt. c expression in a dose-dependent manner, indicating that it affected anticancer drug resistance and induced programmed cell death in cancerous cells. Upon release from the mitochondrial membrane in the cytoplasm, Cyt. c activates procaspase-9 which has a proteolytic activity. ^7^ In the intrinsic apoptosis pathway, procaspase-9 is an initiator caspase that is present in a monomer form and consists of three domains. ^3^ In the event of caspase-9 activation, it cleaves and activates executor caspase-3. It is known that caspase-3, a major component of the execution pathway, cleaves DNAase inhibitors and proteins of the cytoskeleton, causing the fragmentation of cells. ^3, 8^ It is believed that caspase-3 participates in proteolytic series by activating caspases -6, -7, and -9 to facilitate the disintegration of the apoptotic cells prior to their removal by phagocytosis. ^3^ Generally, cell death results from the execution pathway, which is demonstrated by blebbing membranes, DNA disintegration condensed chromosomes, and cell shrinkage. Evidence suggests that deregulation of specific caspases enhances the tumorigenic potential of cells. ^9^

One of the most prominent examples of non-coding RNAs (ncRNAs) are small nucleolar RNAs (snoRNAs). snoRNA is a small RNA molecule that typically consists of 60 to 300 nucleotides and is found primarily in the nucleus of a cell. ^10^ In the late 1970s, snoRNA’s primary role was uncovered in research to ensure that ribosomes, the cellular machinery that synthesizes proteins, are assembled and function correctly. ^11^ In recent years, researchers have covered a wide variety of regulatory processes, including transcription, post-transcriptional modification, and translation. However, it was found that snoRNA was significantly downregulated in meningiomas compared with normal brain tissue. This led to further investigation of the potential role of snoRNA in cancer. ^12^ Clinical samples and cell lines have also shown that snoRNAs are expressed in a variety of malignancies, indicating that they may be used as prognostic and diagnostic indicators for diseases including breast cancer and non-small cell lung cancer (NSCLC). Among the snoRNAs that are frequently overexpressed in breast cancer, prostate cancer, and lung cancer, SNORA42, SNORD15A, SNORD15B, SNORD22, SNORD17, and SNORD87 have been demonstrated to correlate with tumorigenicity, highlighting snoRNA’s significance in regulating cancer biology. ^13^ As demonstrated by the strong correlation between a poor clinical outcome and increased SNORD52 expression in hepatocellular carcinoma (HCC). SNORD52 increases the stability of the CDK1 protein, which in turn promotes the development of HCC. Therefore; targeting the Upf1/SNORD52/CDK1 pathway may have therapeutic potential for the treatment of HCC. ^14^ Though their processes, particularly their functions in cellular signal transduction pathways, are not fully understood, the majority of recent reports focus on SNORAs screening and verifying their correlations with illnesses. Based on these findings, it is possible that SNORAs play an important role at the transcriptional and epigenetic levels, as well as in healthy and tumor tissues and body fluids. ^12, 13^

Small nucleolar RNA, H/ACA box 64 (SNORA64) or U64 is one of the SNORAs that enables protein binding, as evidenced by Inferred Physical Interaction (IPI). In addition, SNORA64 is involved in processing primary RNA transcripts into one or more mature RNA molecules as Inferred from Electronic Annotation (IEA). ^15, 16^ The interaction of this molecule with a number of proteins could be involved in modulating telomerase, according to some researchers. ^15^ Further, it may serve as a potential osteoarthritis biomarker. ^17^ According to our earlier research, there are variations in mRNA expression between the three distinct tumor grades, indicating a significant part for SNORA64 in the genesis and progression of pancreatic cancer. Furthermore, there is a link between the stimulation of the epithelial-to-mesenchymal transition and the reduction of SNORA64 expression. ^18^ Due to the lack of knowledge of SNORAs’ impact on apoptotic pathways, we are currently examining how SNORA64 affects the expression of both pro- and anti-apoptotic genes in pancreatic cancer cells.

## 2. Material and methods

### 2.1 Cell line and construct

Pancreatic cancer cell line PK-8 obtained from Riken Cell Bank (RRID:CVCL_4718). PK-8 with SNORA64 knockdown and scramble cell line as a control are used in this study. The efficiency of the transfection was around 50%, as determined in our previous study. ^18^ A humidified incubator is used for cultivation of cell lines in RPMI-1640 containing 10% fetal bovine serum (FBS), 100 u/ml of penicillin, 100 g/ml of streptomycin, and 5% carbon dioxide.

### 2.2 Real-time reverse transcription polymerase chain reaction (RT-PCR)

Using two sets of primers (left and right), each gene’s RNA expression was measured using RT-PCR analysis with four replicates for each sample. Gel electrophoresis utilizing ethidium bromide was employed for the confirmation of qPCR results. Data for gene sequences were obtained from the National Center for Biotechnology Information (NIH), starting with Homo sapiens tumor suppressor P53. Homo sapiens BCL2 and BCL2-related protein, long isoform (BCL-Xl) have been used as anti-apoptotic gene markers. Homo sapiens BCL2 like 11(BIM), BCL2 antagonist of cell death (BAD), BH3 interacting domain death agonist (BID), BCL2 associated X protein (BAX), and BCL2 antagonist/killer (BAK) have been used as pro-apoptotic genes. Homo sapiens CASP-9, CASP-3, and CASP-7 genes are used as indicators of the initiation of the apoptotic cascade. β-ACTIN, a housekeeping protein, is the reference gene used to normalize the amounts of mRNA ( Table 1). The Trizol technique (MRC, Cata#RT111) was used to extract total RNA, and the nanodrop spectrophotometer used to measure the RNA purity and concentration. cDNA was produced using ProtoScript ^®^ M-MuLV TaqRT-PCR kit (NEB, cat#E6400S). ^18^ The mixture’s templet cDNA’s targeted genes were amplified using Biorad CFX96. ^18, 19^

**Table 1.** Left and right primer sequences used in (RT-PCR) analysis.

Gene symbol	Primer sequences pairs	
	5'-Left Primer Sequence-3'	5'-Right Primer Sequence-3'
P53	aggttggctctgactgtacc	gattctcttcctctgtgcgc
BCL2	ggaggattgtggccttcttt	gccgtacagttccacaaagg
BCL-Xl	catggcagcagtaaagcaag	tcccggaagagttcattcac
BAD	gaagactccagctctgcaga	catcccttcgtcgtcctcc
BID	ggcctaccctagagacatgg	tggctaagctcctcacgtag
BIM	tggcccttttgctaccagat	aggaggacttggggtttgtg
BAX	tctgacggcaacttcaactg	ttgaggagtctcacccaacc
BAK	ccaggacacagaggaggttt	ctctgagtcatagcgtcggt
CASP -9	ctagtttgcccacacccagt	cctttcaccgaaacagcatt
CASP-3	tggaattgatgcgtgatgtt	ggcaggcctgaataatgaaa
CASP -7	ttccacggttccaggctatt	agttccttggtgagcatgga
β-ACTIN	agaaaatctggcaccacacc	agaggcgtacagggatagca

All primers are designed by the author.

### 2.3 Cell proliferation assay

Six-well plates were seeded with 2X10 ^4^ SNORA64 knockdown cells of PK-8, along with scramble cell lines as a control, and incubated for six hours. Trypsin was used to collect the cells, and then medium-washed over them. The Vi-cell XR (cell viability analyzer) uses trypan blue dye to determine the quantity of viability and viable cells.

### 2.4 Analytical statistics

After the data were examined, a two-tailed Student’s t-test was used to identify any significant differences in gene expression. P-value ≤ 0.05.

## 3. Results

### 3.1 Pk-8 SNORA64 knockdown shows a significant low expression of tumor suppressor P53

**Figure 1.**
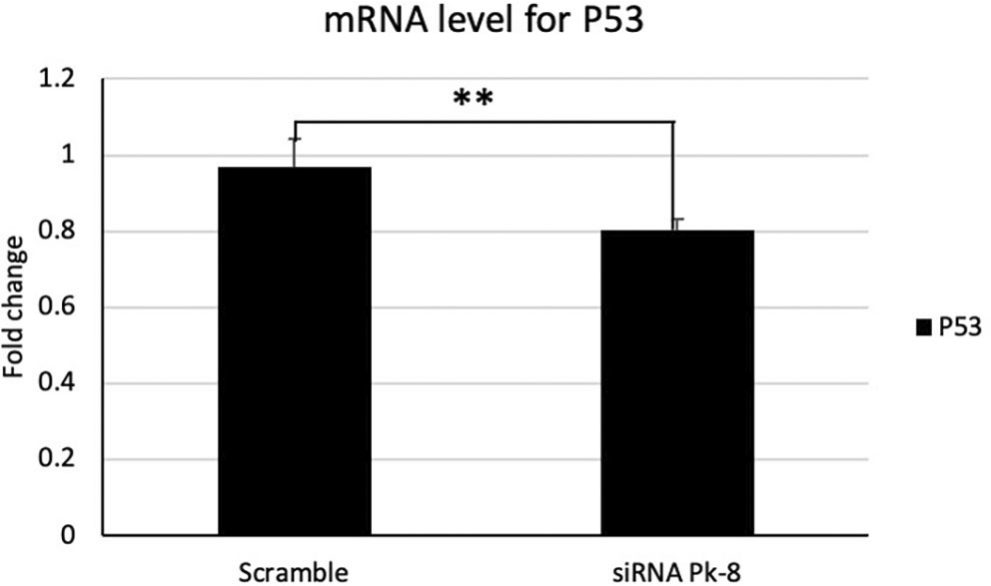
Tumor suppressor P53 mRNA expression level in Pk-8 SNORA64 knockdown cell line. Pk-8 with SNORA64 knockdown significantly downregulated the expression of p53 mRNA compared to the scramble control cell line. P-value was 0.01.

In comparison to a scrambled control cell line, Pk-8 with SNORA64 knockdown significantly downregulated the expression of p53 ( Figure 1).

### 3.2 Pk-8 SNORA64 knockdown shows significant differences in the expression of apoptotic regulator genes

**Figure 2.**
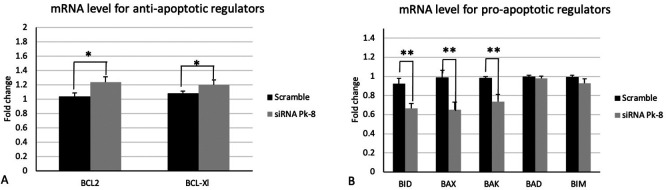
Apoptotic regulators mRNA expression levels in the Pk-8 SNORA64 knockdown cell line. A.Pk-8 with SNORA64 knockdown significantly upregulated the expression of anti-apoptotic regulator genes BCL2 and BCL-Xl in contrast with the scramble. P-value were 0.01, and 0.01respectively. B. In comparison to the scramble control cell line, Pk-8 with SNORA64 knockdown markedly reduced the pro-apoptotic regulator genes BID, BAX, and BAK. BAD and BIM, however, do not differ from scramble cell line control in terms of expression. P-value were 0.001, 0.001, 0.001, 0.1 and 0.06 respectively.

Pk-8 cell line with SNORA64 knockdown significantly upregulated the expression of anti-apoptotic regulator genes BCL2 and BCL-Xl in contrast with the scramble ( Figure 2A).

In comparison to a scrambled control cell line, Pk-8 with SNORA64 knockdown significantly downregulated the pro-apoptotic genes BID, BAX, and BAK ( Figure 2B). Despite this, there is no change in the expression of BAD and BIM in Pk-8 with SNORA64 knockdown compared to the scrambled control cell line ( Figure 2B).

### 3.3 Pk-8 SNORA64 knockdown shows significantly low expression of apoptotic enzymes

**Figure 3.**
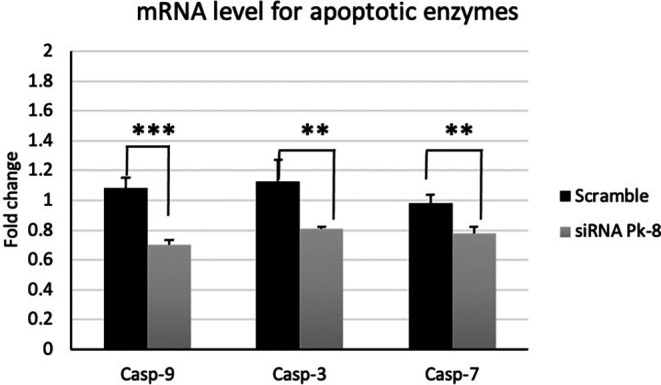
Apoptotic enzyme mRNA expression levels in the Pk-8 SNORA64 knockdown cell line. Pk-8 with SNORA64 knockdown significantly downregulated the expression of apoptotic enzyme regulator genes CASP-9, 3, and 7 compared to the scramble control cell line. P-value were 0.0001, 0.002, and 0.001 respectively.

In comparison to a scrambled control cell line, Pk-8 with SNORA64 knockdown significantly downregulated the expression of apoptotic enzyme genes CASP-9, 3, and 7 ( Figure 3).

### 3.4 Pk-8 SNORA64 knockdown shows significant survival n in viable cells and viability

**Figure 4.**
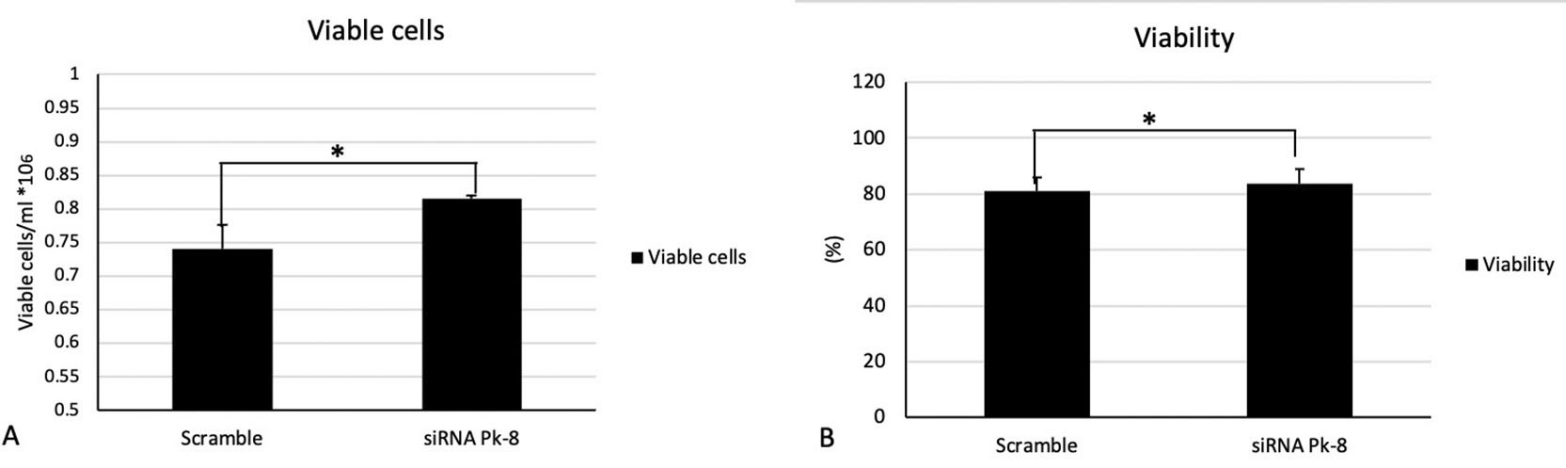
Cellular viabilities in Pk-8 SNORA64 knockdown cell line. A. In comparison with the scramble control cell line, Pk-8 with SNORA64 knockdown showed a significant increase in viable cells. P-value was 0.01. B. Pk-8 with SNORA64 knockdown significantly increased the viability of the cells compared to the scramble control cell line. P-value was 0.01.

In comparison to a scrambled control cell line, Pk-8 with SNORA64 knockdown significantly elevated the number of viable cells ( Figure 4A) and viability ( Figure 4B).

## 4. Discussion

Despite the limited treatment options and poor prognosis for pancreatic cancer, this disease is highly aggressive and deadly. ^1^ One of the characteristics of pancreatic cancer that encourages initiation, growth, and therapeutic resistance is apoptosis avoidance. ^2, 3, 19^ Apoptosis relies on specific signaling pathways that are often deregulated in cancer development and progression. ^2, 3^ The apoptosis pathway is cooperatively coordinated by tumor suppressor p53 signaling pathways. Epidemiological research and molecular evidence have shown that P53 mutations are associated with an increased risk of developing cancer. ^3, 4, 20^ Our results show low expression of P53 in the Pk-8 with SNORA64 low expression compared to the control. It is well known that genetic alteration in the tumor suppressor P53 is common during stages of pancreatic carcinogenesis. ^20^ By interacting with the multidomain members of the BCL2 family, p53 directly contributes to the intrinsic apoptotic pathway by inducing permeabilization of the mitochondrial outer membrane. ^3^ Furthermore, an investigation was conducted to determine how the expression of the p53 and BCL2 proteins relates to the various kinds of human cancers. ^20^ In the malignant tumors, there was a noteworthy negative connection observed between the expression of p53 and BCL2. ^10, 20^ According to our findings, Pk-8 has elevated BCL2 and BCL-XL expression, while SNORA64 expression is low. The BCL2 and BCL-XL dimers prevent death signals and promote cell survival. ^3^ As pancreatic cancer progresses, BCL-XL expression rises. Treatments using anti-BCL-XL may be able to stop pancreatic tumors from progressing from their primary to more advanced stages. ^21^ Furthermore, BCL-XL overexpression raises the incidence rates of pancreatic cancer by preventing apoptosis and senescence brought on by oncogenes. ^22^

It is well known that the tumor suppressor gene p53 essential for maintaining genomic stability. It controls the production of the anti-apoptosis molecule BCL2 as well as the pro-apoptosis molecule BAX. ^4^ The pro-apoptotic gene BAX and the anti-apoptotic gene BCL2 have been found to be significant participants in the control of apoptosis in pancreatic cancer. ^22^ Conversely, this study shows a decline in the expression of the pro-apoptotic molecules BID, BAK, and BAX, while SNORA64 expression is low. BID is one of the molecules that activate BAK and BAX dimerization. BID protein is truncated in the presence of one of the intrinsic signaling apoptosis. Truncated BID can activate the effector proteins BAK and BAX. The BAK and BAX dimers bind to and sequester the anti-apoptotic BCL2 and BCL-XL. ^3, 5^ The subsequent signals have an impact on the potential of the mitochondrial membrane and result in the release of Cyt. c to the cytoplasm. Consequently, the release of Cyt. c and the activation of Casp-9 and 3 initiate the cascade of caspases. ^3, 6^ Casp-3 activation is thought to initiate the apoptotic execution route since it can activate Casp-6 and 7. The execution route is irreversible and constitutes the last phase of apoptosis induction. ^3^ Many studies demonstrated the Casp-3 role in the progression, aggressiveness, and overall survival time of patients with gastric, ovarian, prostate, cervical, and colorectal cancer. ^23– 25^ Furthermore, Casp-3 can suppress dissemination and invasion and promote the epithelial-to-mesenchymal transition phenotype. Casp-3 is therefore regarded as a significant prognostic marker and an indicator for cancer disease-free survival rates as well as overall 5-year survival rates. ^23, 25^ When compared to the scrambled control cells, our data demonstrate a decrease in the key caspases’ mRNA expression in the intrinsic signaling apoptosis Casp-9, 3, and 7. Despite this, SNORA64 knockdown showed no effect on the expression of BAD and BIM in Pk-8 with low expression of SNORA64 when compared with the scrambled control cells.

Pro-apoptotic and anti-apoptotic regulator equilibrium is essential for homeostasis, and any change toward survival through a variety of escape mechanisms will encourage tumor growth. ^3^ One of the primary methods that tumor cells develop resistance to chemotherapy and radiation treatment is through apoptosis-regulatory mediators, particularly elevated concentrations of anti-apoptotic proteins. ^26^ Restoring apoptosis in the tumor cells resistant to chemotherapy and radiation therapy is crucial to the treatment. ^26, 27^ Therefore, SNORA64 can be used as a therapeutic agent to increase cell sensitivity to death by chemotherapy. A future step should be taken to investigate the synergistic effect of SNORA64 and chemotherapy in cancer treatment.

The imbalance of both pro- and anti-apoptotic genes may stimulate tumor growth and progression in pancreatic cancer by increasing cell proliferation, cell viability, and decreasing cell death. ^1, 2^ Our results indicated a significant increase in viable cells and viability in Pk-8 with low expression of SNORA64. Culture productivity is increased when anti-apoptosis genes, such as BCL2, are overexpressed and suppress apoptosis. ^5^ We also recently reported that the expression of SNORA64 in pancreatic cancer tissues is significantly higher than that in normal pancreatic ductal tissues. ^18^ Several studies have demonstrated the critical role played by SNORAs in cancer development and their potential uses as biomarkers and/or therapeutic targets. ^12– 18^ As a result, changes in SNORAs expression could significantly affect the pathogenic processes and pathways underlying the development of cancer. Our research shows that, among particular molecules, the SNORA64 molecule has the capacity to activate the apoptotic intrinsic pathway or enhance sensitivity to death. On the other hand, SNORA64 could be a key indicator for cell proliferation and survival.

## 5. Conclusion

Based on our findings, SNORA64 may modulate both pro-apoptotic and anti-apoptotic genes that control apoptosis. SNORA64 promotes apoptosis by increasing the expression of pro-apoptotic molecules such as BID, BAX, and BAK. Meanwhile, SNORA64 expression impact the anti-apoptosis molecules BCL2 and BCL-XL by decreasing their expression. Consequently, low expression of SNORA64 increases the viability of pancreatic cancer cells by increasing viable cells number.

## Ethics information

This study was completed in accordance with Southern Technical University’s ethical guidelines with approval number 5/1863. This study did not involve human participants.

## Data Availability

I consent to make all of the information and resources used to support the findings or analyses in my research publicly available under an open license that allows CC-BY.4.0 reuse. The datasets with DOI 10.6084/m9.figshare.30742097 are freely available at https://figshare.com. ^28^
