# Supplementary material for: Effect of Small Nucleolar RNA H/ACA Box 64 (SNORA64) on Apoptosis Regulator Genes in Pancreatic Cancer in Vitro
Source: F1000Res. 2026 May 12;15:68. Originally published 2026 Jan 16. [Version 3] doi: 10.12688/f1000research.173746.3 (PMC13090758; doi:10.12688/f1000research.173746.3)
Supplement: Peer review history

# Reviewer report: Reviewer response for version 3

**DOI:** 10.5256/f1000research.200550.r483982

**Published:** 2026-05-23

**Authors:** Dheyaa Al-Sallami

**Affiliations:** 1 Pharmacology, Southern Illinois University School of Medicine, Springfield, Illinois, USA

I have reviewed the revised version of the manuscript and the authors’ responses. The revisions have adequately addressed my previous concerns, and I have no further comments to add.

Is the work clearly and accurately presented and does it cite the current literature?

Yes

If applicable, is the statistical analysis and its interpretation appropriate?

Yes

Are all the source data underlying the results available to ensure full reproducibility?

Yes

Is the study design appropriate and is the work technically sound?

Yes

Are the conclusions drawn adequately supported by the results?

Yes

Are sufficient details of methods and analysis provided to allow replication by others?

Yes

Reviewer Expertise:

Molecular Oncology, CRISPR design and application, Molecular Biology, Molecular Neuroscience ,

I confirm that I have read this submission and believe that I have an appropriate level of expertise to confirm that it is of an acceptable scientific standard.

# Reviewer report: Reviewer response for version 2

**DOI:** 10.5256/f1000research.197739.r471178

**Published:** 2026-04-20

**Authors:** Dheyaa Al-Sallami

**Affiliations:** 1 Pharmacology, Southern Illinois University School of Medicine, Springfield, Illinois, USA

The paper addresses a genuinely underexplored question — the role of a specific H/ACA snoRNA in pancreatic cancer apoptosis — and the direction is scientifically interesting. However, the study has significant methodological and interpretive gaps that must be addressed before full approval.

 The following specific corrections are requested. I would be prepared to approve the manuscript after these revisions have been satisfactorily addressed

 Here are the 11 unique comments:

**1. Title**: Replace "Small Nuclear RNA 64" with "Small Nucleolar RNA, H/ACA Box 64" to reflect the correct molecular classification per HGNC nomenclature.

**2. Abstract**, line 1: Same nomenclature correction: "Small Nuclear RNA" to be "Small Nucleolar RNA." Also, add a sentence briefly defining the H/ACA snoRNA family for readers unfamiliar with ncRNA biology.

**3. Abstract, Results:** The abstract summarizes results as directional changes without quantification. Include representative fold-change values and p-values for key findings (e.g., p53, BCL2, CASP-3) to strengthen the abstract's informativeness.

**4. Introduction, paragraph 2: **The introduction would benefit from a brief overview of H/ACA snoRNA biology: canonical pseudouridylation function, emerging non-canonical roles, and why SNORA64 specifically was selected for study.

**5. Methods, RT-PCR: **Specify whether this is semi-quantitative RT-PCR or quantitative real-time RT-PCR (qRT-PCR).

**6. Methods, Statistics: **Clarify whether n = 4 refers to biological replicates (independent experiments) or technical replicates.

**7. Results, Cell Viability: **Clearly state the time points at which viability was measured. Distinguish between viability (live/dead) and proliferation in the text.

**8. Discussion, page 7: **Correct "pro-apoptotic molecules BCL2 and BCL-XL" to "anti-apoptotic molecules BCL2 and BCL-XL." This is a factual error.

**9. Discussion, Mechanism: **Add a paragraph discussing possible mechanisms by which a snoRNA could influence mRNA levels of apoptosis genes. Consider: (1) rRNA pseudouridylation affecting translational fidelity, (2) snoRNA-derived small RNAs with miRNA-like activity, (3) direct snoRNA-protein interactions. Acknowledge that the current study cannot distinguish among these possibilities.

**10. Discussion, Therapeutic: **Remove or substantially qualify the claim that "SNORA64 can be used as a therapeutic agent." This requires in vivo data, pharmacokinetic studies, and drug combination experiments that are entirely absent from this work.

**11. Conclusion: **Rewrite to use correlative language: "SNORA64 knockdown was associated with..." rather than "SNORA64 promotes apoptosis by increasing..." Distinguish clearly between observation and interpretation.

Is the work clearly and accurately presented and does it cite the current literature?

Yes

If applicable, is the statistical analysis and its interpretation appropriate?

Yes

Are all the source data underlying the results available to ensure full reproducibility?

Yes

Is the study design appropriate and is the work technically sound?

Yes

Are the conclusions drawn adequately supported by the results?

Yes

Are sufficient details of methods and analysis provided to allow replication by others?

Yes

Reviewer Expertise:

Molecular Oncology, CRISPR design and application, Molecular Biology, Molecular Neuroscience ,

I confirm that I have read this submission and believe that I have an appropriate level of expertise to confirm that it is of an acceptable scientific standard, however I have significant reservations, as outlined above.

# Reviewer report: Reviewer response for version 2

**DOI:** 10.5256/f1000research.197739.r471177

**Published:** 2026-04-17

**Authors:** Ramina Khoshaba Elia

**Affiliations:** 1 University of Baghdad, Baghdad, Baghdad Governorate, Iraq

Dr. Al-Fardan addresses an important and emerging topic in cancer biology, particularly the role of non-coding RNAs in apoptosis with clear biological focus on intrinsic apoptosis pathway. The study explores the role of SNORA64, a small nucleolar RNA, in regulating apoptosis-related genes in pancreatic cancer using an in vitro model. Given the increasing importance of non-coding RNAs in cancer biology, 

**Title:**

 I suggest revising the title to better highlight the outcomes of the study. For example: **"SNORA64 Knockdown Promotes Anti-Apoptotic Signaling and Cell Survival in Pancreatic Cancer Cell"**

**2. Materials and Methods**

 The gene expression analysis provides useful information, it does not confirm whether apoptosis is actually occurring. Techniques such as Annexin V/PI staining, caspase activity assays, or flow cytometry should be included to validate the biological effects.

**2.2 Real-time reverse transcription polymerase chain reaction (RT-PCR).**

 - The qPCR analysis lacks information on normalization methods (e.g., ΔΔCt). Also, Primer validation and efficiency are not reported .

 - The use of gel electrophoresis for qPCR validation is no longer commonly used.

**2.4 Analytical statistics**

 The use of Student’s *t*-test alone may not be sufficient given the number of comparisons performed across multiple genes. The application of more appropriate statistical methods, such as analysis of variance (ANOVA) followed by post hoc testing, is recommended.

**Writing and Presentation:**

 The manuscript is generally well structured; however, there are multiple language and grammar issues that affect clarity. Professional editing services are recommended to address language issues.

Is the work clearly and accurately presented and does it cite the current literature?

Yes

If applicable, is the statistical analysis and its interpretation appropriate?

Partly

Are all the source data underlying the results available to ensure full reproducibility?

Yes

Is the study design appropriate and is the work technically sound?

Partly

Are the conclusions drawn adequately supported by the results?

Partly

Are sufficient details of methods and analysis provided to allow replication by others?

Yes

Reviewer Expertise:

Genetics, Cancer Research, Molecular Biology, Microbial Genetics, Gene Expression and qPCR Analysis.

I confirm that I have read this submission and believe that I have an appropriate level of expertise to confirm that it is of an acceptable scientific standard.

# Reviewer report: Reviewer response for version 2

**DOI:** 10.5256/f1000research.197739.r470979

**Published:** 2026-04-10

**Authors:** Tingbo Liang

**Affiliations:** 1 Zhejiang University School of Medicine, Hangzhou, Zhejiang, China

The authors have responded appropriately to the issues raised, and I consider the manuscript acceptable in its revised form.

Is the work clearly and accurately presented and does it cite the current literature?

Yes

If applicable, is the statistical analysis and its interpretation appropriate?

Partly

Are all the source data underlying the results available to ensure full reproducibility?

Yes

Is the study design appropriate and is the work technically sound?

Partly

Are the conclusions drawn adequately supported by the results?

Partly

Are sufficient details of methods and analysis provided to allow replication by others?

Yes

Reviewer Expertise:

NA

I confirm that I have read this submission and believe that I have an appropriate level of expertise to confirm that it is of an acceptable scientific standard.

# Reviewer report: Reviewer response for version 1

**DOI:** 10.5256/f1000research.191586.r455858

**Published:** 2026-02-23

**Authors:** Tingbo Liang

**Affiliations:** 1 Zhejiang University School of Medicine, Hangzhou, Zhejiang, China

Alfardan examines SNORA64 knockdown in PK-8 pancreatic adenocarcinoma cells using qPCR of BCL-2 family genes and viability/proliferation assays to argue SNORA64 regulates intrinsic apoptosis. While the concept is interesting, the conclusions are based mainly on mRNA changes and non-specific viability readouts, without direct apoptosis validation and with insufficient methodological detail. Substantial additional experiments and clearer reporting are required.

1. Apoptosis is inferred from mRNA only; functional apoptosis assays are needed. Please include at least one direct apoptosis readout:  Annexin V/PI (or Annexin V/7-AAD) flow cytometry;Caspase-3/7 activity, cleaved caspase-3 and cleaved PARP by Western blot;Mitochondrial membrane potential (JC-1/TMRE) and cytochrome c release to connect to intrinsic pathway. Without these, it is difficult to conclude that apoptosis is truly altered rather than changes in transcription accompanying altered growth state.
2. Clarify causality and strengthen mechanistic claims. The conclusion states “SNORA64 interactions with apoptotic inhibitor molecules,” but no interaction data are described. If “interaction” is meant mechanistically, consider: RNA pulldown / RIP (e.g., with candidate RNA-binding proteins) or CLIP-based approaches;Assessment of snoRNA canonical function (rRNA modification targets) and whether those changes affect translation of apoptosis regulators. 
3. Use of a single cell line limits generalizability. Findings are based on PK-8 only. Pancreatic cancer is heterogeneous. Please validate in additional PDAC lines (e.g., PANC-1, MiaPaCa-2, BxPC-3, AsPC-1) and/or a non-malignant pancreatic ductal cell line as a baseline.
4. Knockdown validation and experimental design details are insufficient. Provide SNORA64 knockdown efficiency (qPCR Ct values, % reduction) and number of independent biological replicates.
5. Therapeutic sensitization claim requires treatment context. The final sentence implies SNORA64 can increase sensitivity “during treatment,” but no drug or stress condition is tested. Consider adding experiments with standard PDAC agents (e.g., gemcitabine, nab-paclitaxel, irinotecan/5-FU regimens) or apoptosis inducers, showing differential response between scramble and knockdown (or rescue with SNORA64 re-expression).

Is the work clearly and accurately presented and does it cite the current literature?

Yes

If applicable, is the statistical analysis and its interpretation appropriate?

Partly

Are all the source data underlying the results available to ensure full reproducibility?

Yes

Is the study design appropriate and is the work technically sound?

Partly

Are the conclusions drawn adequately supported by the results?

Partly

Are sufficient details of methods and analysis provided to allow replication by others?

Yes

Reviewer Expertise:

Pancreatic cancer

I confirm that I have read this submission and believe that I have an appropriate level of expertise to state that I do not consider it to be of an acceptable scientific standard, for reasons outlined above.

# Response

**Published:** 2026-03-13

**Authors:** Rana Alfardan

**Affiliations:** Community health Department, Southren Technical University, Basrah, Basrah, Iraq

Dear Dr. Tingbo Liang,

 Thank you very much for your time and for your insightful comments on my manuscript. I would like to address the points you have raised and provide clarification where necessary:

1. Regarding the assessment of apoptosis, quantitative real-time PCR (qPCR) results provide supporting evidence of apoptosis through the evaluation of changes in gene expression. Nevertheless, I acknowledge that confirmation via protein cleavage assays or flow cytometry constitutes an essential step and will be pursued in future investigations.
2. The use of a single cell line is acknowledged as a limitation of the current study. This point has now been explicitly mentioned in the second version of the manuscript.
3. Transfection of the PK-8 cell line was carried out using siRNA oligonucleotides delivered via the psiRNA-h7SKG1 vector. The specific oligo sequences and the detailed protocol are provided in the cited reference. In accordance with the other reviewer’s suggestion, the type of small RNA employed for knockdown has now been clearly specified in the second version.
4. With respect to the therapeutic sensitization effect, I would like to clarify that this interpretation remains speculative at this stage and is proposed as a direction for future studies.

Thank you again for your valuable feedback, which has helped strengthen the manuscript.

 Sincerely,

 Dr. Rana Alfardan

# Reviewer report: Reviewer response for version 1

**DOI:** 10.5256/f1000research.191586.r451315

**Published:** 2026-01-21

**Authors:** Adeolu Oluremi

**Affiliations:** 1 University of Arkansas at Little Rock, Little Rock, Arkansas, USA

**Title & Abstract**

**Title:** Consider making it more specific and impactful.

**Suggestion:** *"Downregulation of SNORA64 Promotes Pancreatic Cancer Cell Survival by Modulating the Apoptotic Regulator Gene Network"*

**Abstract**

**Methods:** The methods description is overly brief. Specify the key techniques used (e.g., RT-qPCR for gene expression, cell viability assay using trypan blue exclusion).

**Results:** Include the key finding about p53 downregulation, as it's a central result. Mention the lack of change in BAD/BIM for completeness.

**Conclusion:** State the potential therapeutic implication more clearly (e.g., "SNORA64 may serve as a sensitizer to apoptosis-inducing therapies.").

**Introduction**

**Flow:** The transition from general apoptosis mechanisms to snoRNAs to SNORA64 specifically is somewhat abrupt. The rationale for studying *SNORA64 in pancreatic cancer* needs a stronger link.

**Suggestion:** After introducing snoRNAs in cancer, add 1-2 sentences summarizing your previous study (mentioned in the Abstract) that identified SNORA64 as a predictive marker in pancreatic cancer, which directly motivates the current functional investigation into apoptosis.

**Materials & Methods**

**Clarity and Detail:**

**2.1:** Specify how the SNORA64 knockdown was achieved (e.g., shRNA, siRNA) and the source/sequence of the scramble control.

**2.2:** The description of gel electrophoresis to "confirm qPCR results" is unusual, as qPCR is typically quantitative and agarose gels are qualitative. Clarify if this was for product verification.

**Table 1:** The table formatting is corrupted in the text. Ensure it is presented clearly with separate columns for Gene Symbol, Left Primer, and Right Primer.

**2.4:** Mention the software used for statistical analysis.

**Results**

**Logical Order:** The results for p53 (3.1) are critical and should be presented before the BCL2 family results (3.2), as p53 is a key upstream regulator of many of these genes. This improves the narrative flow.

**Figure References:** Ensure every figure (1, 2A/B, 3, 4A/B) is explicitly called out in the results text in sequential order.

**Language:** Consistently use "knockdown" or "low expression." Avoid switching between them (e.g., "Pk-8 with SNORA64 knockdown" vs. "Pk-8 with low expression of SNORA64").

**Discussion**

**Structure:** The discussion is comprehensive but can be better organized to mirror the results.

**Paragraph 1:** Discuss p53 downregulation and its implications.

**Paragraph 2:** Discuss the BCL2 family (anti-apoptotic: BCL2, BCL-XL; pro-apoptotic: BID, BAX, BAK) results.

**Paragraph 3:** Discuss the caspase results and their connection to the BCL2 pathway.

**Paragraph 4:** Synthesize how these molecular changes explain the increased cell viability.

**Paragraph 5:** Discuss the lack of effect on BAD/BIM, which is an interesting negative result—speculate briefly on why SNORA64 might not affect these specific BH3-only proteins.

**Mechanistic Speculation:** The discussion excellently connects the dots. To strengthen it, propose a testable hypothetical model in a sentence (e.g., "We propose that SNORA64 may influence a transcriptional or post-transcriptional regulator that coordinately modulates p53 and a subset of BCL2 family genes, but not BAD/BIM.").

**Limitations:** Add a brief limitations subsection before the conclusion. Acknowledge that this is an *in vitro* study using a single cell line (PK-8). Future work should validate findings in other pancreatic cancer cell lines and *in vivo* models.

Is the work clearly and accurately presented and does it cite the current literature?

Yes

If applicable, is the statistical analysis and its interpretation appropriate?

Partly

Are all the source data underlying the results available to ensure full reproducibility?

Yes

Is the study design appropriate and is the work technically sound?

Yes

Are the conclusions drawn adequately supported by the results?

Yes

Are sufficient details of methods and analysis provided to allow replication by others?

Yes

Reviewer Expertise:

Cancer Biology, Infectious diseases, Nanotechnology, Biomedical Sciences

I confirm that I have read this submission and believe that I have an appropriate level of expertise to confirm that it is of an acceptable scientific standard, however I have significant reservations, as outlined above.

# Response

**Published:** 2026-03-12

**Authors:** Rana Alfardan

**Affiliations:** Community health Department, Southren Technical University, Basrah, Basrah, Iraq

**Subject: Revision of the research article (Effect of Small Nuclear RNA 64 (SNORA64) on Apoptosis Regulator Genes in Pancreatic Cancer in Vitro)**

 Dear Dr. Adeolu Oluremi,

 Thank you very much for your thoughtful and constructive suggestions. I have accepted all of them, and they have significantly enriched my manuscript. Please find below a point-by-point response to your comments:

1. **Title:** Thank you for your valuable suggestion regarding the manuscript title. I agree that the proposed title, **"Downregulation of SNORA64 Promotes Pancreatic Cancer Cell Survival by Modulating the Apoptotic Regulator Gene Network,"** would indeed be powerful and appropriate for this work.
  However, upon further consideration of this modification, I have encountered a significant practical constraint. The data of the manuscript has already been published online under its current title, making a title change procedurally difficult at this stage. Furthermore, the previous two editors involved with this manuscript explicitly endorsed the current title during the review process.
  Given these circumstances, and with respect for the editorial consistency maintained throughout the review process, I believe the title should remain unchanged. I hope you understand this decision, and I appreciate your understanding regarding this limitation.
  Sincerely,
2. **Abstract:** As suggested, the abstract has been revised to specify the key techniques used. It now also explicitly includes the key findings regarding p53 downregulation and the lack of change in BAD/BIM. Furthermore, the potential therapeutic implications have been stated more clearly.
3. **Conclusion:** The conclusion has been revised exactly as you suggested.
4. **Introduction:** Our previous study is already mentioned in the introduction. The text has been revised to directly introduce pancreatic cancer and explicitly motivate the current functional investigation into apoptosis, creating a smoother transition from the background to the study's objective.
5. **Materials and Methods:**
  - **2.1:** The transfection of the PK-8 cell line is performed with siRNA oligos carried by psiRNA-h7SKG1. The oligo sequences and the protocol are detailed in the cited reference. However, as you suggested, the type of small RNA used for the knockdown has now been specified in the text.
  - **2.2:** The sentence regarding the use of agarose gels for qPCR product verification has been revised as you suggested for clarity.
  - **Table 1:** The table format has been changed as you recommended. It now consists of three columns: Gene Symbol, Left Primer, and Right Primer.
  - **2.4:** Excel has now been added to the "Materials" subsection as one of the software used for statistical analysis.
6. **Results:**
  - The results for p53 (3.1) are now presented before the BCL2 family results (3.2), following the logical flow you recommended.
  - **Figure References:** All figures (Figure 1, 2A/B, 3, 4A/B) are now explicitly called out in the results text in sequential order.
  - **Language:** For consistency, the phrasing "PK-8 with SNORA64 knockdown" is now used consistently throughout the manuscript.
7. **Discussion:**
  - **Paragraph 1:** Two sentences have been added to discuss the observed downregulation of p53 and its potential implications.
  - **Paragraph 2:** This paragraph now focuses on discussing the BCL2 family, including both pro- and anti-apoptotic molecules.
  - **Paragraph 3:** Two sentences have been added to discuss the connection between the caspase cascade and the BCL2 pathway.
  - **Paragraph 4:** This paragraph has been rephrased, and three sentences have been added to elaborate on the increase in cell viability.
  - **Paragraph 5:** A new paragraph specifically discussing BIM and BAD has been added.
  - **Mechanistic Speculation:** Thank you for this excellent suggestion. This section has been incorporated into the revised manuscript (version 2).
8. **Limitations:** A new "Limitations" subsection has been added to the discussion, as you recommended, to address the constraints of the current study.

Thank you again for your time and expertise, which have greatly improved the quality of my work.

 Sincerely,

 Dr. Rana Alfardan
